# Hypoxia‐induced cofilin 1 promotes hepatocellular carcinoma progression by regulating the PLD1/AKT pathway

**DOI:** 10.1002/ctm2.366

**Published:** 2021-03-21

**Authors:** Bowen Yao, Yazhao Li, Tianxiang Chen, Yongshen Niu, Yufeng Wang, Yuanyuan Yang, Xinyu Wei, Qingguang Liu, Kangsheng Tu

**Affiliations:** ^1^ Department of Hepatobiliary Surgery The First Affiliated Hospital of Xi'an Jiaotong University Xi'an China; ^2^ Center for Translational Medicine The First Affiliated Hospital of Xi'an Jiaotong University Xi'an China; ^3^ Xi'an Jiaotong University Health Science Center Xi'an China

**Keywords:** CFL1, hepatocellular carcinoma, hypoxic microenvironment, PLD1

## Abstract

**Background:**

Hepatocellular carcinoma (HCC) is the fourth fatal malignant tumour type worldwide. However, the exact molecular mechanism involved in HCC progression remains unclear.

**Methods:**

Three pairs of HCC and matched portal vein tumour thrombus (PVTT) tissue samples were analysed by isobaric tags for relative and absolute quantification (iTRAQ) assay to investigate the differentially expressed proteins. Real‐time quantitative PCR, immunostaining, and immunoblotting were performed to detect cofilin 1 (CFL1) in HCC and non‐tumour tissues. CCK8 and EdU, and Transwell assays, respectively, determined cell proliferation, migration, and invasion of HCC cells. Further, subcutaneous and tail vein injection were performed in nude mice for investigating HCC growth and lung metastasis in vivo. Regulatory effect of hypoxia‐inducible factor‐1α (HIF‐1α) on CFL1 was confirmed by chromatin immunoprecipitation (ChIP) assay. Finally, interaction between CFL1 and phospholipase D1 (PLD1) was studied using immunoprecipitation (IP) assay.

**Results:**

The iTRAQ analysis identified expression of CFL1 to be significantly upregulated in PVTT than in HCC tissues. Increased expression of CFL1 was closely associated with unfavourable clinical features, and was an independent risk predictor of overall survival in HCC patients. The knockdown of CFL1 inhibited cell growth viability, invasiveness, and epithelial‐mesenchymal transformation (EMT) in HCC cells. Furthermore, CFL1 silencing significantly suppressed the growth and lung metastasis of HCC cells in nude mice. Next, HIF‐1α directly regulated CFL1 transcription by binding to the hypoxia‐responsive element (HRE) in the promoter. Moreover, we disclosed the interaction between CFL1 and PLD1 in HCC cells using IP assay. Mechanistically, CFL1 maintained PLD1 expression by repressing ubiquitin‐mediated protein degradation, thereby activating AKT signalling in HCC cells. Notably, the CFL1/PLD1 axis was found mediating the hypoxia‐induced activation of the AKT pathway and EMT.

**Conclusion:**

The analysis suggests that hypoxia‐induced CFL1 increases the proliferation, migration, invasion, and EMT in HCC by activating the PLD1/AKT pathway.

## Graphical abstract

1. Cofilin 1 is overexpressed in HCC, especially in PVTT tissues.
2. Cofilin 1 is up‐regulated in hypoxia condition and promotes HCC progression.
3. CFL1 binds to PLD1 and maintains PLD1 protein stability.
4. HIF‐1α regulates CFL1 transcription and activates CFL1‐PLD1‐AKT axis in HCC.

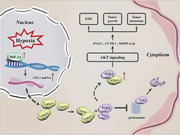

**Abbreviations**
ADF: actin depolymerizing factor
CFL1: cofilin 1
ChIP: chromatin immunoprecipitation
ERK1/2: extracellular signal‐regulated kinases 1 and 2
ESFTs: Ewing's sarcoma family tumours
GEO: Gene Expression Omnibus
HCC: hepatocellular carcinoma
HDACi: histone deacetylases inhibitor
HIF‐1α: hypoxia inducible factor 1 α
HRE: hypoxia response element
iTRAQ: isobaric tags for relative and absolute quantification
KEGG: the Kyoto Encyclopedia of Genes and Genomes
LIMK: LIM domain kinase
MAPK: mitogen‐activated protein kinas
MMPs: matrix metalloproteinases
PHD: prolyl hydroxylase
PLD1: phospholipase D1
PVTT: portal vein tumour thrombus
TCGA: the Cancer Genome Atlas
VHL: Von Hippel‐Lindau tumour suppressor

## 1 INTRODUCTION

Hepatocellular carcinoma (HCC), one of the most common tumours worldwide,^1^ ranks 4th in the incidence and 2nd in cancer‐related deaths in China.^2^ Eighty per cent of the HCC patients are initially diagnosed in the middle or late stages, thereby missing the opportunity for radical treatments.^3^ Intrahepatic and extrahepatic metastases are the primary reason for high mortality and poor prognosis in HCC.^4^ However, the molecular mechanism involved in HCC progression is not elucidated. Thus, it is necessary to elucidate the exact mechanism underlying HCC growth and metastasis and identify novel therapeutic targets to improve patients' prognoses.

Hypoxia is an essential feature of HCC microenvironment.^5^ The median partial pressure of oxygen in HCC tissues is only 6 mmHg, while that in normal liver tissues is 30 mmHg.^6^ Hypoxia stabilizes hypoxia‐inducible factors (HIFs), which are heterodimers composed of HIF‐1/2α subunit and constitutively expressed HIF‐1β subunit.^7^ With oxygen as the substrate, the HIF‐1/2α subunit is hydroxylated by prolyl hydroxylase (PHDs) and recognized by von Hippel‐Lindau (VHL) tumour suppressor for ubiquitin‐mediated proteolysis.^8^ Under hypoxic conditions, HIF‐1/2α is translocated into nucleus and forms a transcriptional complex with HIF‐1β and transcriptional coactivators, thereby activating target gene transcription by binding to the hypoxia‐responsive elements (HREs, 5′‐A/GCGTG‐3′) on DNA.^9^ Our previous studies have revealed several hypoxia‐responsive genes, including vasodilator‐stimulated phosphoprotein (VASP), long non‐coding RNA (lncRNA) EIF3J‐AS1, tuftelin 1 (TUFT1), miR‐3677‐3p/miR‐3682‐3p, regulating HCC growth and metastasis.^10^, ^11^, ^12^, ^13^, ^14^ Liu et al. confirmed that hypoxia induced beta‐catenin overexpression promoted in vitro invasion and in vivo metastasis of MHCC97 and Hep3B cells.^15^ Some researchers found p300 was highly expressed in HCC specimens and activated the translocation of β‐catenin into the nuclei, increased cyclin D1 activity and enhanced the migration/invasion of HCC cells.^16^

Cofilin 1 (CFL1) is a subtype of actin‐depolymerizing factor (ADF)/cofilin family proteins, which plays a crucial role in tissue developments, internal environment homeostasis, and diseases.^17^, ^18^ CFL1 plays a critical role in controlling timing, turnover, and F‐actin assembly dynamics inside daughter cell nuclei.^19^ Moreover, the class for LIM kinase (LIMK) family phosphorylation of cofilin/ADF proteins is depended on the LIMK1: CFL1 co‐crystal structure.^20^ In colorectal cancer (CRC), knockdown of CFL1 represses cell migration, invasion, and EMT in cancer cells via regulation of actin cytoskeleton organization.^21^ CFL1 is frequently overexpressed in pancreatic cancers, and its increased level in the serum indicate poor prognosis in patients.^22^ CFL1 is highly expressed in endometrial cancer, and its knockdown reduces the invasive ability and matrix metalloproteinases activity in cancer cells.^23^ CFL1 is also recognized as a tumour‐promoting factor in Ewing's sarcoma family tumours (ESFTs) cell proliferation and metastasis.^24^ The upregulated expression of CFL1 has been reported in a previous study.^25^ The expression of CFL1 is induced by hepatitis B virus X protein (HBx) and is frequently found to be upregulated in tissue samples from HBV‐related HCC patients.^26^ Furthermore, CFL1 is highly expressed in histone deacetylase inhibitor (HDACi)‐resistant HCC cells, and is phosphorylated by activated extracellular signal‐regulated kinases 1 and 2 (ERK1/2).^27^ However, biological role of CFL1 and its underlying mechanism in HCC under a hypoxic microenvironment is still unclear.

Phospholipase D(PLD) hydrolyses phosphatidylcholine to form phosphatidic acid and choline.^28^ The two most typical PLD subtypes, PLD1 and PLD2, are essential to maintain cells or intima and involve in various cellular biological processes, such as cell growth, proliferation, migration, cytoskeleton recombination, and intracellular protein transport.^29^, ^30^, ^31^ Moreover, PLD family proteins promote tumour growth and metastasis in gastric cancer, colorectal cancer, prostate cancer, and breast cancer.^28^, ^32^, ^33^, ^34^ The PLDs may also contribute to cell invasion and cancer metastasis, secretion of matrix metalloproteinases (MMPs), and EMT.^35^, ^36^ PLD1 induces EMT and activation of integrin family pathway, followed by activation of downstream mitogen‐activated protein kinase(MAPK, also known as p38), extracellular regulated genes 1(Erk1), and Akt (also known as protein kinase B).^29^, ^35^ However, no report explains the relationship between CFL1 and PLD1 in cancer.

In this study, proteomics analysis reported that CFL1 was highly expressed in PVTT than that in matched HCC tissues. Moreover, CFL1 in HCC was determined using our data and publicly available databases. Necessary experiments (in vitro and in vivo) were performed to investigate the role of CFL1 in HCC cell growth and metastasis. Furthermore, we found that CFL1 was a hypoxia‐responsive gene, and it mediated hypoxia‐induced HCC progression by regulating the PLD1/AKT pathway.

## 2 MATERIALS AND METHODS

### 2.1 Patients and tissue samples

One hundred pairs of tumour and adjacent non‐tumour tissues were obtained from HCC patients who underwent hepatectomy. None of the patients in the First Affiliated Hospital of Xi'an Jiaotong University, whose specimens were identified as HCC by pathologists received any non‐surgical treatment before radical resection. Three paired PVTT and HCC were subjected to iTRAQ assay for investigating the differentially expressed proteins. All quick‐frozen tissue samples in liquid nitrogen were stored at −80°C. Moreover, Table 1 lists the detailed clinical features of HCC patients.

**TABLE 1:** Correlation between the clinicopathologic characteristics and CFL1 expression in hepatocellular carcinoma

			CFL1 expression		
Characteristics		*n *= 100	Low (*n *= 50)	High (*n *= 50)	*p*
Age (year)	<50	34	15	19	0.398
	≥50	66	35	31	
Gender	Male	84	40	44	0.275
	Female	16	10	6	
HBV infection	Absent	22	16	6	0.016*
	Present	78	34	44	
Serum AFP level (ng/ml)	<20	34	20	14	0.205
	≥20	66	30	36	
Tumour size (cm)	<5	38	25	13	0.013*
	≥5	62	25	37	
Tumour number	1	77	43	34	0.033*
	≥2	23	7	16	
Cirrhosis	Absent	30	19	11	0.081
	Present	70	31	39	
Venous infiltration	Absent	58	38	20	<0.001*
	Present	42	12	30	
Edmondson‐Steiner grading	I+II	63	34	29	0.300
	III+IV	37	16	21	
TNM stage	I+II	82	47	35	0.002*
	III+IV	18	3	15	

Abbreviations: AFP, alpha‐fetoprotein; HBV, hepatitis B virus; TNM, tumour‐node‐metastasis.

* Indicates statistically significant.

### 2.2 Cell culture and transfection

HCCLM3, MHCC97H, Huh7, Hep3B, and HepG2 (some of the classic human HCC cell lines), and a normal hepatic cell line L‐02 were obtained from the Cell Bank of the Chinese Academy of Sciences (Shanghai, China) and cultured with complete DMEM medium at standard conditions, as previously described.^37^ Incubators with 1% O_2_ to mimic a hypoxic microenvironment were prepared for cells in hypoxia group. CFL1 shRNAs (shRNA1, shRNA2, and shRNA3), nontargeting (NT) shRNA, CFL1 overexpression plasmid (Ev‐D0115‐Lv105), and PLD1 overexpression plasmid (Ev‐Z6402‐Lv237) were provided by GeneCopoeia. Furthermore, we purchased HIF‐1ɑ siRNA, PLD1 siRNA, and NT siRNA from GenePharma. Cell transfection was performed using Oligofectamine™ Transfection Reagent and Lipofectamine™ Stem Transfection Reagent, according to the manufacturer's protocols (both kits were the production of Thermo Fisher Scientific (Waltham, MA, USA)). The sequences of shRNAs and siRNAs are presented in Table S1.

### 2.3 Real‐time quantitative PCR (RT‐qPCR)

Total RNA (tissues or cells) were extracted by TRIzol. Then, cDNA reversed from RNA was obtained followed by protocols of cDNA synthesis kits. Details and primers are described in the Supporting Information.

### 2.4 Cell proliferation analysis

Cell viability was detected via the Enhanced Cell Counting Kit‐8 (Beyotime, Shanghai, China) and 5‐ethynyl‐2′‐deoxyuridine (EDU) Labeling/Detection Kit (Ribobio, Guangzhou, China). Details are described in the Supporting Information.

### 2.5 Transwell assay

The Transwell chamber coated with or without Matrigel was prepared for cell invasion or migration assays. Details are described in the Supporting Information.

### 2.6 Western blotting

RIPA lysis buffer (Beyotime) was used for total protein extraction from cells and tissues. The concentration of the protein sample was determined and tests were based on standard western blotting procedures. Details of antibodies and protocols of blotting are listed in the Supporting Information.

### 2.7 Tumour xenograft experiments

Animal experiments approved by the Institutional Animal Care and Use Committee of Xi'an Jiaotong University included two models, subcutaneous and tail vein injection models. Thirty male BALB/C mice have been raised in the specific pathogen free (SPF) animal experimental facility. After one‐week‐feeding, four weeks old mice were subcutaneously injected with 3 × 10^6^ HCCLM3 or MHCC97H cells with or without a CFL1 knockdown at the left flanks (5 mice in each group). The mice's weight and tumour size were measured every seven days. The mice were sacrificed on the 28th day, and the subcutaneous tumours were harvested for immunohistochemistry (IHC) staining. As previously mentioned, eighteen male mice were injected with 1 × 10^6^ HCCLM3 or MHCC97H cells via the lateral tail vein (3 mice in each group). On the sixth week, mice in these group were sacrificed and the lung tissues were collected for further experiments, such as IHC and hematoxylin‐eosin (H&E) staining, as previously described.^38^

### 2.8 IHC staining

Paraffin‐embedded sections of tissues underwent dewaxing, hydration, and antigen retrieval. Then, endogenous peroxidase was blocked by 3% H_2_O_2_. Tissue sections were incubated with antibodies. Further steps and details are listed in the Supporting Information.

### 2.9 Other methods

Other methods and materials used in this study are presented in the Supporting Information, such as chromatin immunoprecipitation (ChIP), co‐immunoprecipitation (co‐IP), luciferase reporter assay and statistical analysis.

## 3 RESULTS

### 3.1 CFL1 is highly expressed in HCC

**FIGURE 1:**
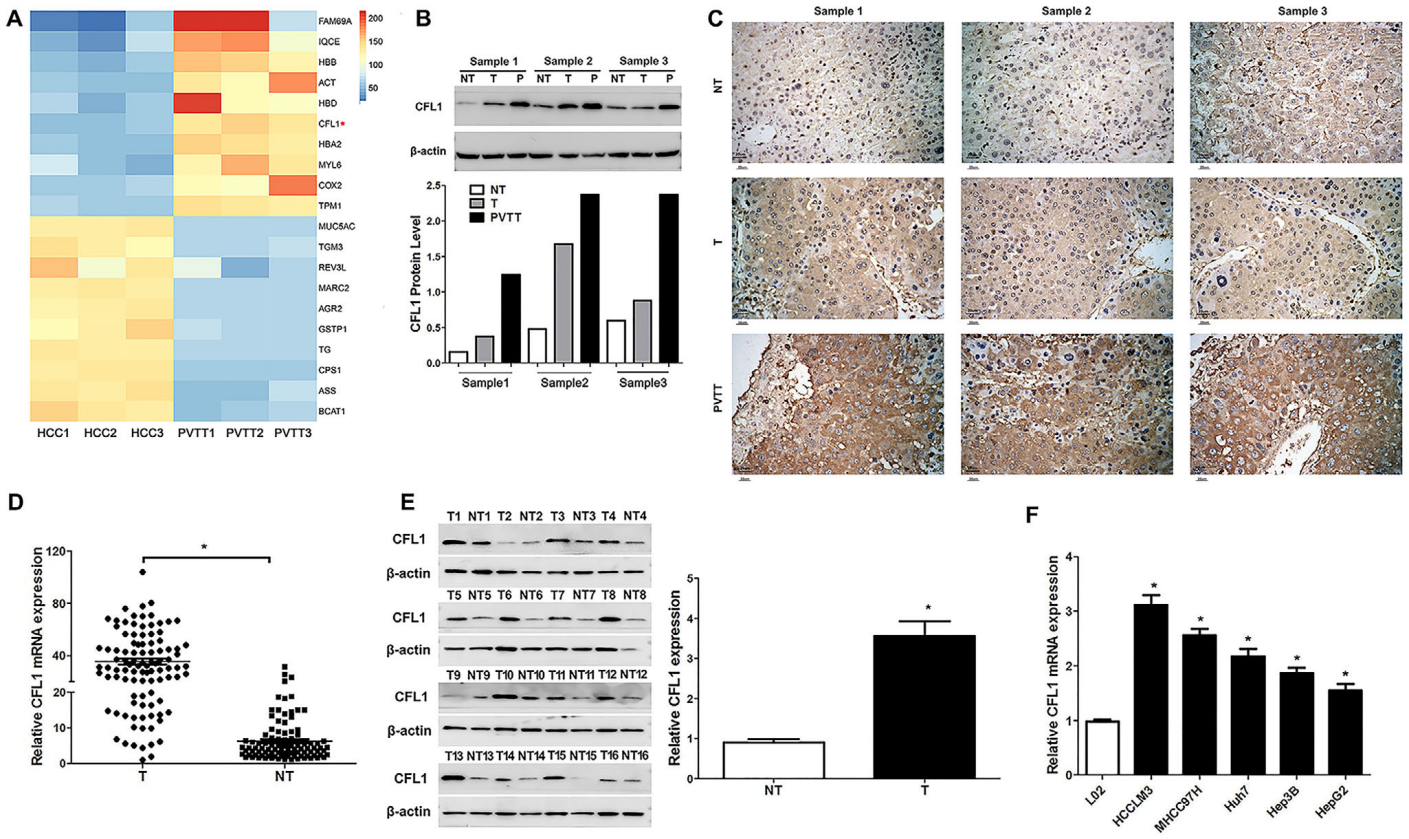
The expression of CFL1 among PVTT, HCC, and adjacent non‐tumour tissues. (A) The heat map of the top ten upregulated/downregulated proteins in PVTT compared to matched HCC tissues. (B) Western blotting analysis of CFL1 was performed among PVTT, HCC, and tumour‐adjacent tissues. (C) IHC staining of CFL1 was performed among PVTT, HCC, and tumour‐adjacent tissues. (D) RT‐qPCR results indicated that CFL1 mRNA expression was significantly higher in HCC than that in tumour‐adjacent tissues. (E) Sixteen pairs of HCC and matched tumour‐adjacent tissues were subjected to immunoblotting for CFL1 expression. (F) The CFL1 mRNA levels in five HCC cell lines (HCCLM3, MHCC97H, Huh7, Hep3B, and HepG2) and a normal hepatic cell line (L02).T: HCC, NT: non‐tumour tissue, P: PVTT. *p < 0.05

PVTT is a critical risk factor for poor prognosis in HCC.^28^ Three pairs of HCC and matched PVTT tissue samples were analysed by iTRAQ assay to investigate the differentially expressed proteins. According to the screening criteria (fold change > 1.2 and *p* < 0.05), 946 differentially expressed proteins were recognized (Table S2). Of the top ten upregulated proteins (Figure 1A), CFL1 was highly expressed in HCC than normal liver tissues, according to the Cancer Genome Atlas (TCGA) and Gene Expression Omnibus (GEO) (Figure S1A,B). Western blotting and IHC analysis consistently indicated a gradually upregulated expression of CFL1 in tumour‐adjacent, HCC, and PVTT tissues (Figure 1B,C). Further, 100 pairs of collected HCC and non‐tumour tissues were subjected to RT‐qPCR and immunoblotting for CFL1 expression. The results further demonstrated that CFL1 expression in HCC was significantly higher than that in tumour‐adjacent tissues (Figure 1D,E). Moreover, upregulated expression of CFL1 was also detected in HCC cell lines we mentioned previously than that in L‐02 foetal hepatocyte cells (Figure 1F).

### 3.2 Elevated levels of CFL1 confer to the poor prognosis in HCC

Next, we analysed the correlation between CFL1 expression and clinical parameters in HCC. HCC patients were distributed into CFL1 high or low expression group as per expression cut‐off value, which was the median mRNA expression in this cohort. As shown in Table 1, increased expression of CFL1 was observed in HCC patients infected with HBV (*p* = 0.016), tumour diameter ≥5 cm (*p* = 0.013), multiple tumours (*p* = 0.033), vascular invasion (*p* < 0.001), and advanced Tumor‐Nodes‐Metastasis stage (*p* = 0.002). Survival analysis indicated patients in the CFL1‐high group showed worse overall survival (OS) than those in the CFL1‐low group (*p* < 0.01, Figure S1C). The TCGA data analysis using GEPIA webtool^29^ confirmed that high CFL1 expression predicted reduced OS in HCC patients (Figure S1D). Moreover, univariate analysis showed that tumour size, tumour number, TNM stage, venous infiltration, HBV infection, and CFL1 level were significantly associated with HCC patients’ OS (Table 2). What is more, multivariate analysis showed that only tumour size, tumour number, venous infiltration, and CFL1 level are independent prognostic indicators of OS in HCC (Table 2).

**TABLE 2:** Univariate and multivariate Cox hazard analysis of clinical features associated with overall survival in hepatocellular carcinoma patients

	Univariate analysis			Multivariate analysis		
Clinical variables	HR	95% CI	*p* value	HR	95% CI	*p* value
Age (≥50 vs. <50)	1.699	0.864‐3.339	0.124	–	–	–
Gender (male vs. female)	1.642	0.942‐2.862	0.080	–	–	–
Tumour size (≥5 vs. <5 cm)	1.717	1.003‐2.938	0.049*	1.534	1.087‐2.165	0.015*
Tumour number (≥2 vs. 1)	3.489	1.631‐7.465	0.001*	2.370	1.479‐3.799	<0.001*
Edmondson stage (III+IV vs. I+II)	1.486	0.452‐4.881	0.514	–	–	–
TNM stage (III+IV vs. I+II)	3.671	1.743‐7.732	0.001*	1.510	0.938‐2.429	0.09
Venous infiltration (present vs. absent)	1.839	1.069‐3.164	0.028*	1.677	1.174‐2.397	0.005*
Serum AFP level (≥20 vs. <20 ng/ml)	1.250	0.760‐2.056	0.380	–	–	–
Cirrhosis (present vs. absent)	0.629	0.360‐1.096	0.102	–	–	–
HBV infection (present vs. absent)	1.820	1.053‐3.146	0.032*	1.534	1.134‐2.823	0.085
CFL1 level (high vs. low)	2.268	1.309‐3.930	0.003*	1.505	1.025‐2.211	0.037*

Abbreviations: CI, confidence interval; HR, hazard ratio.

* Indicates statistically significant.

### 3.3 CFL1 promotes cell proliferation, migration, and invasion in HCC

**FIGURE 2:**
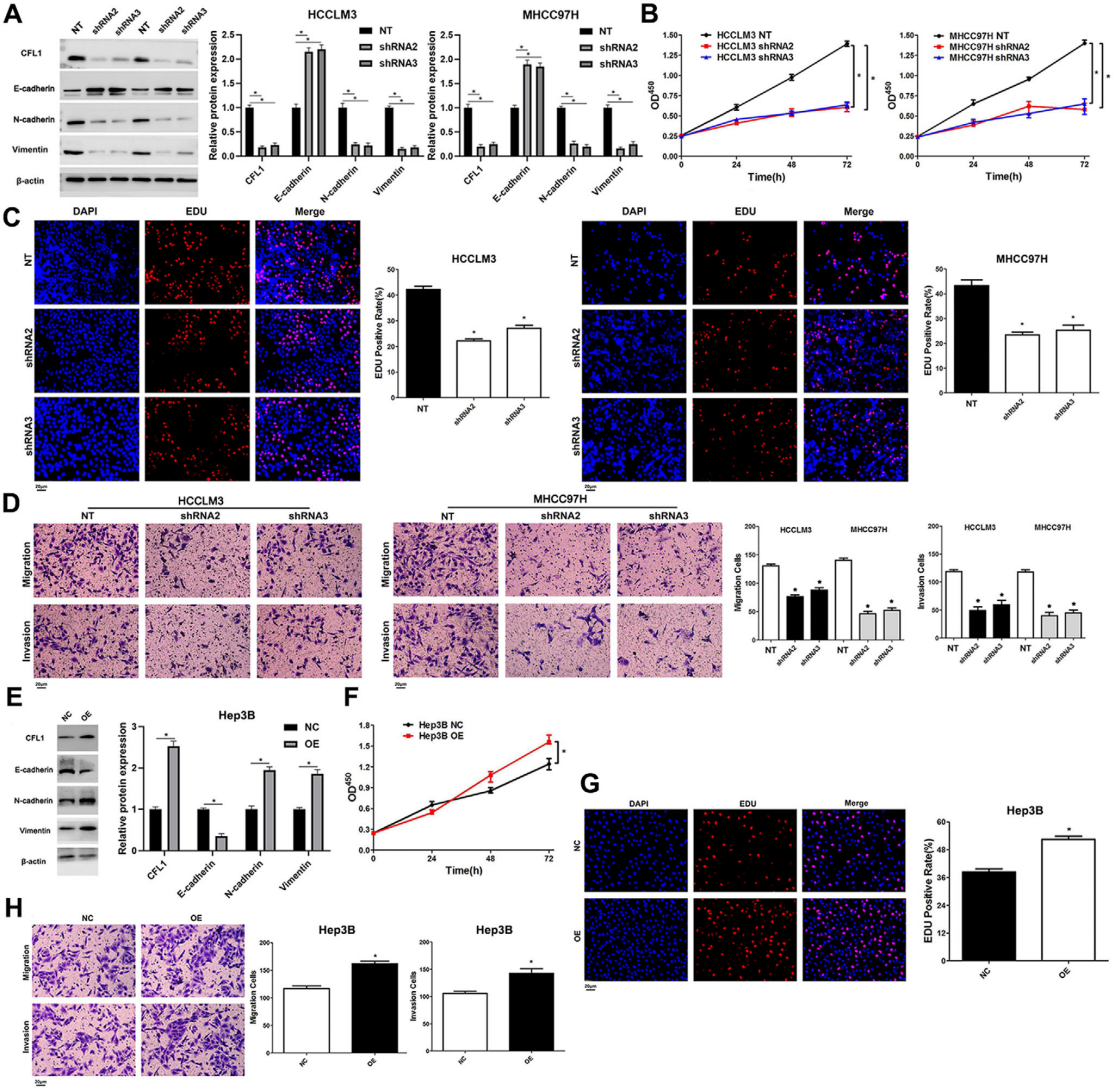
CFL1 promotes the proliferation and invasion of HCC cells. (A) HCCLM3 and MHCC97H cells that were transfected with nontargeting (NT) shRNA or CFL1 shRNAs (shRNA2 and shRNA3) were subjected to western blotting for CFL1, E‐cadherin, N‐cadherin, and Vimentin expression. (B) CFL1 knockdown repressed the viability of HCC cells. (C) CFL1 silencing suppressed the proliferation of HCC cells. (D) HCC cell migration and invasion were reduced by CFL1 knockdown in vitro. (E) Hep3B cells that were transfected with negative control (NC) or CFL1 overexpression plasmid (OE) were subjected to western blotting for CFL1, E‐cadherin, N‐cadherin, and Vimentin expression. (F) CFL1 overexpression promoted the viability of Hep3B cells. (G) Ectopic expression of CFL1 enhanced the proliferation of Hep3B cells. (H) The migration and invasion abilities of Hep3B cells were increased by CFL1 overexpression in vitro. *p < 0.05

CFL1 expression was deleted in HCCLM3 and MHCC97h cells, which highly expressed basal CFL1 (Figure 2A). Cell viability was presented by CCK‐8 and EdU assays, whose results revealed that knockdown of CFL1 prominently suppressed HCC cells’ proliferation (Figure 2B,C). Transwell assays indicated that the migration and invasion potentials of HCC cells decreased while silencing CFL1 expression (Figure 2D). Moreover, CFL1 silencing led to increase in EMT markers such as E‐cadherin, and decrease in some markers such as N‐cadherin and Vimentin (Figure 2A). Conversely, CFL1 was overexpressed in Hep3B cells, which expressed low basal levels of CFL1 (Figure 2E). We found that CFL1 over‐expression markedly promoted cell proliferation, migration ability, invasiveness and EMT in Hep3B cells (Figure 2E‐H).

### 3.4 Effects of CFL1 knockdown on tumour growth and metastasis of HCC in vivo

**FIGURE 3:**
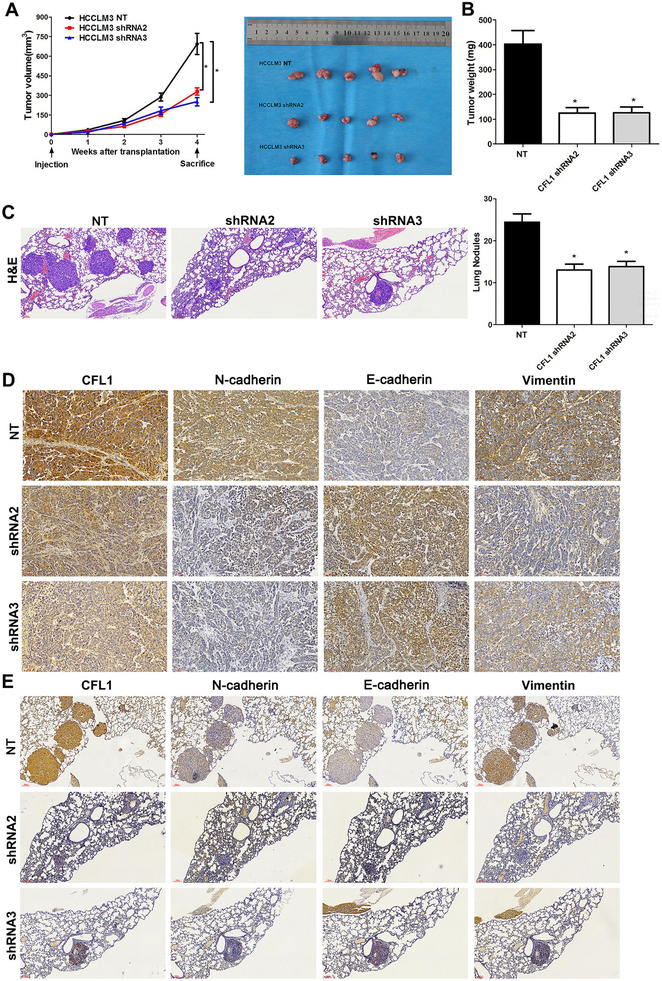
CFL1 knockdown represses HCC growth and lung metastasis in mice. (A) HCCLM3 cells that were transfected with nontargeting (NT) shRNA or CFL1 shRNAs (shRNA2 and shRNA3) were subcutaneously injected into nude mice. The average tumour volume in the CFL1 knockdown group was prominently smaller than the control group. (B) The tumour weights were compared between the CFL1 knockdown group and the control group. (C) HCCLM3 cells with or without CFL1 knockdown were injected into nude mice via the tail vein. H&E staining of lung tissues indicated that CFL1 knockdown reduced lung metastasis of HCC cells. (D) Subcutaneous tumour tissues were subjected to IHC staining for CFL1, E‐cadherin, N‐cadherin, and Vimentin expression. (E) Lung metastases were subjected to IHC staining for CFL1, E‐cadherin, N‐cadherin, and Vimentin expression. *p < 0.05

To confirm the role of CFL1 in vivo, the subcutaneous tumour formation and lung metastasis models in nude mice were established. HCCLM3 cells with or without CFL1 knockdown were subcutaneously injected (within same counted cell number we mentioned before) into nude mice. As shown in Figure 3A,B, CFL1 knockdown remarkably reduced the volume and weight of tumours formed by HCCLM3 cells (*p* < 0.05). Next, we injected HCCLM3 cells with or without a CFL1 knockdown via tail veins to develop a lung metastasis mouse model. Consistent with our hypothesis, metastasis of HCCLM3 cells to the lungs in mice was prominently suppressed by silencing expression of CFL1 (Figure 3C). Furthermore, IHC staining of CFL1, EMT markers (E‐cadherin, N‐cadherin, and Vimentin) was performed in subcutaneous tumour tissues and lung metastases. It was found that CFL1, N‐cadherin, and vimentin levels were reduced while E‐cadherin expression was increased in tumour tissues (CFL1 knockdown group) than in those from controlled tissues (Figure 3D,E). Otherwise, the inhibitory effects of CFL1 knockdown on cancer cell growth and metastasis in HCC were also confirmed in MHCC97H cells (Figure S2).

### 3.5 CFL1 is a downstream target of HIF‐1α in HCC cells

**FIGURE 4:**
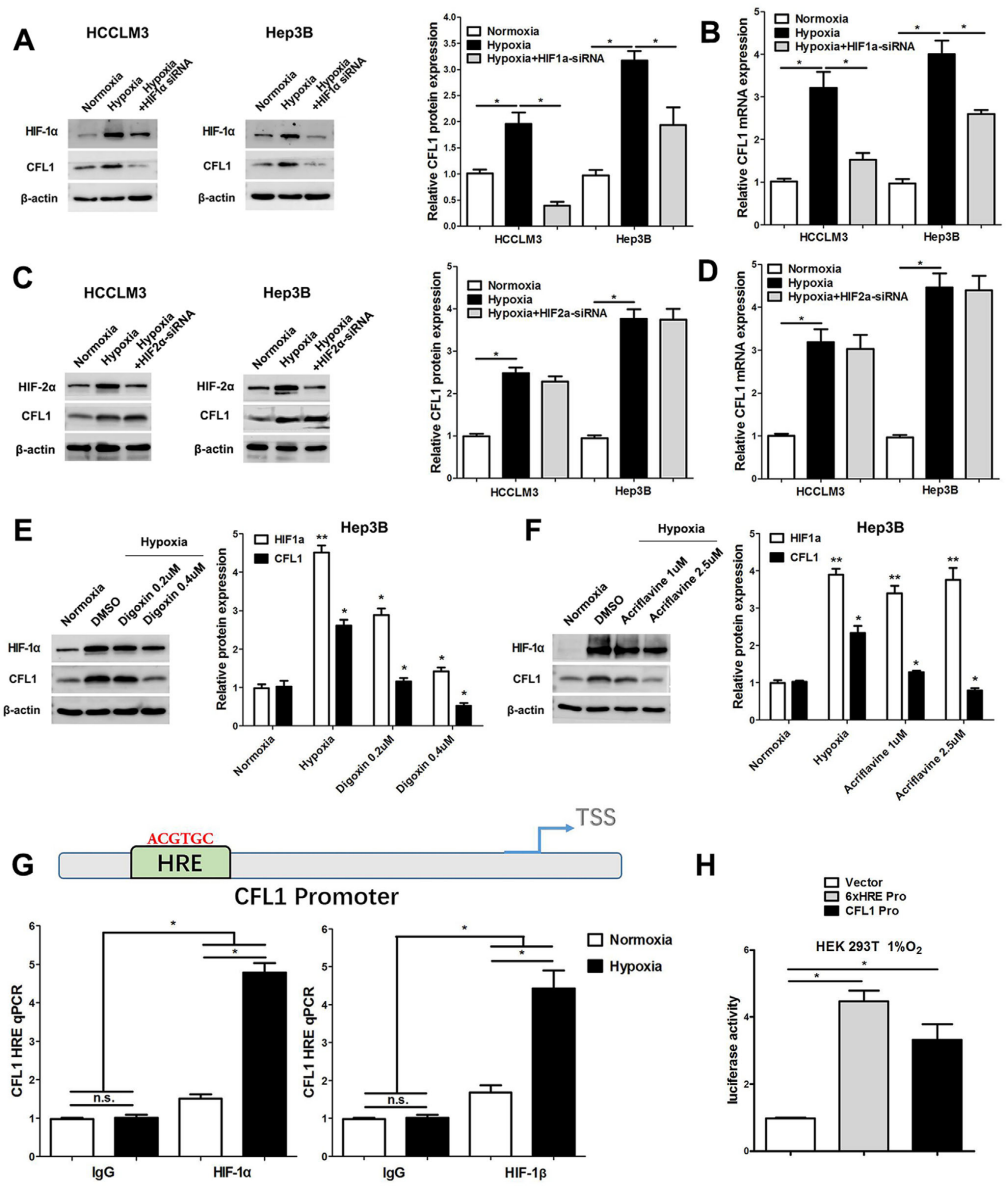
HIF‐1α activates CFL1 transcription in HCC cells. (A) CFL1 protein expression was upregulated in HCCLM3 and Hep3B cells under hypoxic conditions, which could be abolished by HIF‐1α knockdown. (B) HIF‐1α silencing reversed hypoxia‐induced CFL1 mRNA expression in HCC cells. (C) CFL1 protein expression was upregulated in HCCLM3 and Hep3B cells under hypoxic conditions, which could not be abolished by HIF‐2α knockdown. (D) HIF‐2α silencing could not reverse hypoxia‐induced CFL1 mRNA expression in HCC cells. (E,F) HIF‐1α inhibitors (digoxin and acriflavine) consistently reduced the CFL1 level in HCC cells under hypoxic conditions. (G) ChIP assay revealed that HIF‐1α and HIF‐1β directly bond to the CFL1 promoter in HCC cells. (H) HRE luciferase activities were promoted in 293T cells transfected with HRE‐luciferase‐plasmid in hypoxic condition. *p < 0.05

**FIGURE 5:**
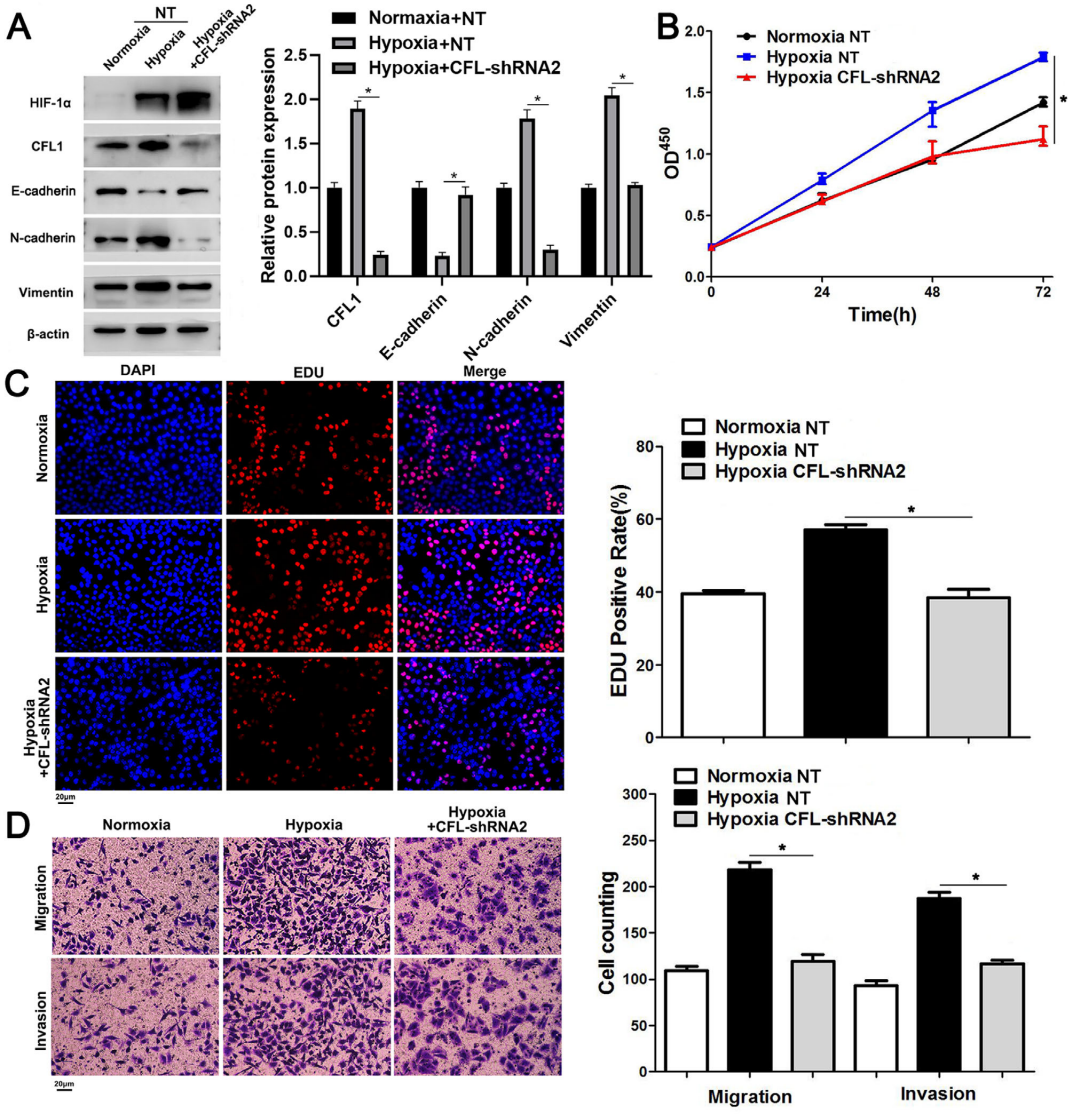
CFL1 knockdown reverses hypoxia‐induced HCC cell proliferation and invasion. (A) HCCLM3 cells that were transfected with nontargeting (NT) shRNA or CFL1 shRNA were cultured in hypoxic conditions. Western blotting analysis was performed to detect HIF‐1α, CFL1, E‐cadherin, N‐cadherin, and Vimentin levels. (B) CFL1 knockdown repressed the viability of HCCLM3 under hypoxic conditions. (C) CFL1 silencing abolished hypoxia‐induced HCCLM3 cell proliferation. (D) The promoting effects of hypoxia on cell migration and invasion were reversed by CFL1 knockdown in HCCLM3 cells. *p < 0.05

To clarify the effect of hypoxia on CFL1 expression in HCC, HCCLM3 and Hep3B cells were cultured in a hypoxic incubator for 48 h. RT‐qPCR and western blotting results demonstrated that hypoxia led to increased CFL1 expression in HCC cells (Figure 4A,B). HIF‐1α is the primary transcription factor, which regulates expression of hypoxia‐responsive genes. Interestingly, hypoxia‐induced CFL1 expression was markedly abolished in HCC cells while HIF‐1α was silencing (Figure 4A,B). However, when HIF‐2α was deleted in HCCLM3 and Hep3B cells and the cells were incubated under hypoxia condition, CFL1 protein or mRNA levels changed without statistical significance (Figure 4C,D). Moreover, treatment with HIF‐1α inhibitors (digoxin and acriflavine) consistently reduced CFL1 levels in HCC cells under hypoxic conditions (Figure 4E,F). The HRE sequences were found in the promoter of CFL1 based on Genomatix and JASPAR databases (Figure S3A). ChIP‐PCR assay further revealed that HIF‐1α and HIF‐1β are directly bound to the HRE in the CFL1 promoter in HCC cells (Figure 4G). Luciferase reporter activities were promoted in HEK 293T cells transfected with HREs‐luciferase‐plasmids or CFL1‐promoter‐luciferase‐plasmids under hypoxic condition (Figure 4H). Next, CFL1 expression was knockdown in HCCLM3 and Hep3B cells under hypoxic conditions (Figure 5A and Figure S4). Our results suggested that CFL1 knockdown significantly reversed hypoxia‐induced cell proliferation, cell migration and invasion, and EMT in HCCLM3 cells (Figure 5A‐D and Figure S4A‐4D). Taken together, these results indicated that CFL1 is transcriptionally regulated by HIF‐1α and mediates hypoxia‐induced HCC progression.

### 3.6 CFL1 maintains PLD1 expression by inhibiting ubiquitin‐mediated degradation

**FIGURE 6:**
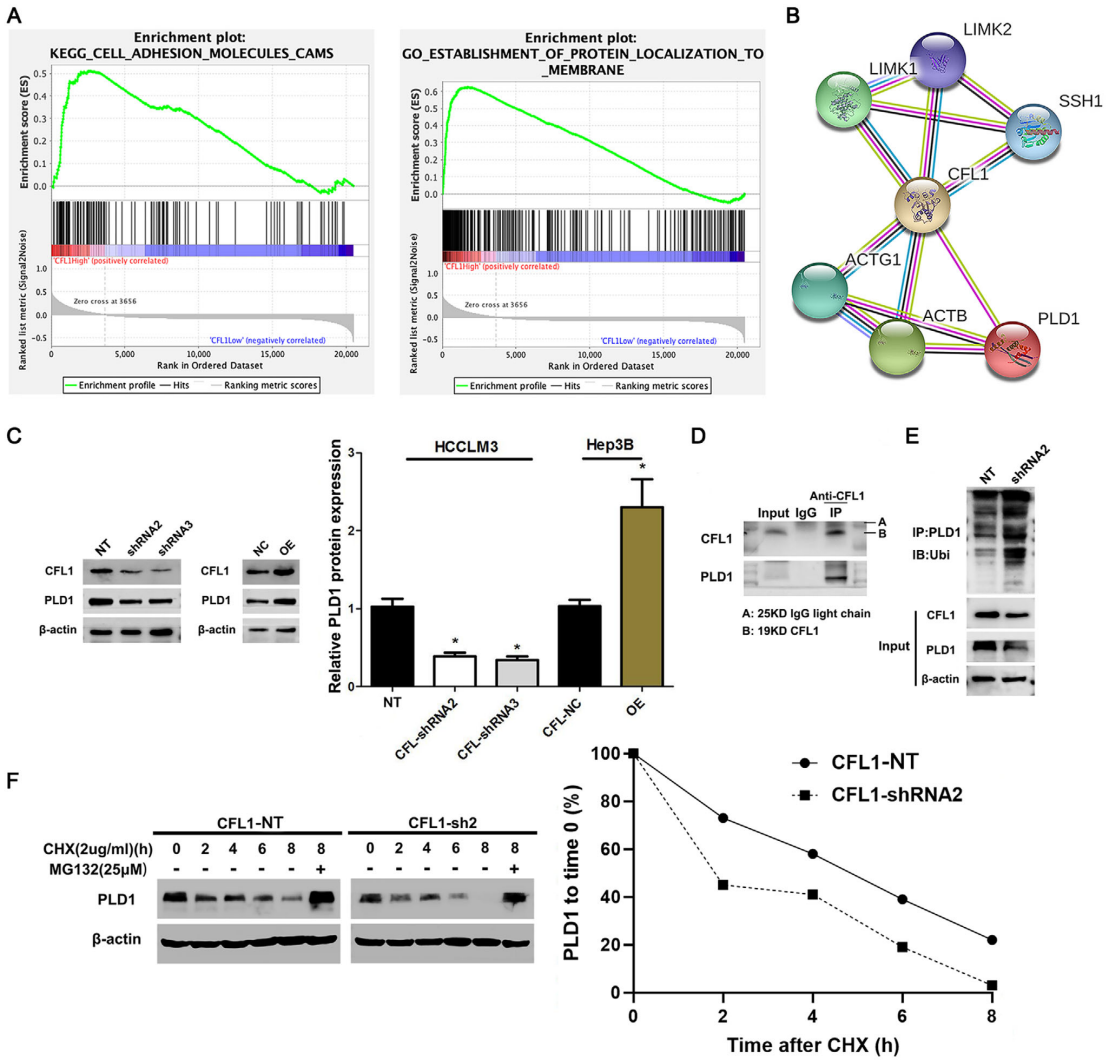
CFL1 regulates PLD1 degradation in HCC cells. (A) KEGG pathway analysis indicated that high expression of CFL1 was closely related to the localization of cell adhesion molecules and proteins located in cell membranes. (B) PLD1 was predicted as a potential CFL1 interactor using the protein interaction database (https://string‐db.org/). (C) HCCLM3 and Hep3B cells were transfected with CFL1 shRNAs (shRNA2 and shRNA3) and CFL1 overexpression plasmid (OE), respectively. Western blotting results indicated that CFL1 positively regulated the PLD1 protein level in HCC cells. (D) Co‐IP assay confirmed the interaction between CFL1 and PLD1 in HCC cells. (E) CFL1 knockdown resulted in increased ubiquitination levels of PLD1 in HCC cells. (F) CFL1 knockdown promoted PLD1 degradation, which could be abolished by MG132 treatment in HCC cells. NT: nontargeting. *p < 0.05

To further explore the downstream mechanism involved in the oncogenic role of CFL1 in HCC, we screened the pathways affected by CFL1 using the Kyoto Encyclopedia of Genes and Genomes (KEGG) database. We argued that high expression of CFL1 was closely related to the localization of cell adhesion molecules and proteins in cell membranes (Figure 6A). Then, we predicted a potential interaction between CFL1 and PLD1 based on the protein interaction database (Figure 6B). CFL1 knockdown reduced protein level of PLD1 in HCCLM3 cells, while CFL1 overexpression enhanced PLD1 protein expression in Hep3B cells (Figure 6C). However, modulating the CFL1 level did not impact PLD1 mRNA expression in HCC cells (Figure S3B). The co‐IP assay demonstrated that CFL1 interacted with PLD1 and its knockdown enhanced the ubiquitination of PLD1 in HCC cells (Figure 6D,E). We used cycloheximide (CHX, 2 μg/ml) to block protein synthesis in HCC cells. Protein degradation curve was drawn to elucidate that PLD1 protein degraded faster when CFL1 was knockdown (Figure 6F). Moreover, treatment of proteasome inhibitor MG132 (25 μM) blocked CFL1‐knockdown‐induced PLD1 degradation in HCC cells (Figure 6F). Subcutaneous tumour tissues from the CFL1 knockdown group showed less PLD1 staining than those from the control group (Figure S5). Thus, these results suggest that CFL1 prevents ubiquitin‐mediated proteolysis of PLD1 in HCC cells.

### 3.7 CFL1/PLD1 axis mediates hypoxia‐induced AKT signalling in HCC cells

**FIGURE 7:**
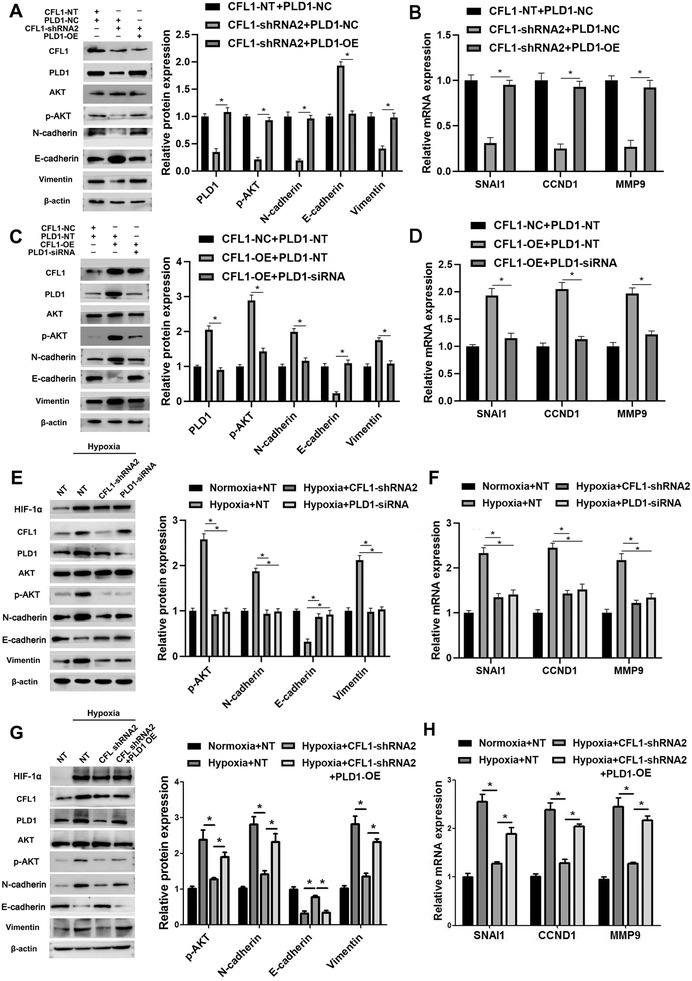
CFL1/PLD1 axis mediates hypoxia‐induced AKT pathway activation in HCC cells. (A) HCCLM3 cells were transfected with indicating vectors, respectively. CFL1 knockdown reduced the p‐AKT level, which could be rescued by PLD1 re‐expression in HCCLM3 cells. (B) CFL1 knockdown reduced the mRNA levels of SNAI1, CCND1, and MMP9, which could be increased by PLD1 restoration in HCCLM3 cells. (C,D) Hep3B cells were transfected with indicating vectors, respectively. PLD1 knockdown reversed CFL1‐induced AKT pathway activation and EMT process in Hep3B cells. (E,F) HCCLM3 cells transfected with CFL1 shRNA or PLD1 siRNA were cultured in hypoxic conditions. Either CFL1 or PLD1 knockdown abolished hypoxia‐induced AKT pathway activation and EMT process in HCCLM3 cells. (G,H) Overexpressed PLD1 in HCCLM3 cells could reverse the inhibition on phosphorylated AKT or EMT process caused by CFL1‐shRNA in hypoxic condition. NT: nontargeting. *p < 0.05

A previous research showed that PLD1 activated AKT and its downstream mammalian target of rapamycin (mTOR) pathways to promote HCC cell proliferation, and invasion.^30^ Consistent with this, we observed that the CFL1 knockdown reduced p‐AKT level, which was enhanced by PLD1 restoration in HCCLM3 (Figure 7A,C). Moreover, PLD1 silencing reversed CFL1‐induced activation of AKT signalling pathway in Hep3B cells (Figure 7B,D). Significantly, either CFL1 or PLD1 knockdown repressed AKT pathway activation and EMT process induced by hypoxia in HCC cells (Figure 7E,F). Recovery experiments were established to investigate the function of PLD1 in HIF‐1α‐CFL1‐PLD1 axis. Overexpressed PLD1 in HCCLM3 cells could reverse the inhibition on phosphorylated AKT or EMT process caused by CFL1‐shRNA in hypoxic condition (Figure 7G,H). Collectively, we confirm that the CFL1/PLD1 axis plays an essential role in hypoxia‐induced HCC progression.

## 4 DISCUSSION

CFL1 is reported to be an over‐expressed gene in HCC, and confers chemo‐resistance in cancer cells to HDAC inhibitors.^26^, ^27^ However, whether CFL1 shows any clinical significance for HCC behaviors remains unclear. Compared to tumour‐adjacent liver tissues, CFL1 expression was prominently increased in HCC tissues, especially was higher in PVTT. The elevated expression of CFL1 was associated with unfavourable characteristics of HCC, including HBV infection, tumour diameter ≥5 cm, multiple tumours, venous infiltration, and advanced TNM stage. Analysis data and TCGA information consistently indicated that HCC patients with high CFL1 expression had worse OS than those who had lower CFL1 expression. Importantly, CFL1 level was an independent prognostic indicator for OS. Thus, these results suggested that CFL1 could be a predicting indicator for HCC patients' prognosis.

The expression of CFL1 is induced by HBx and mainly upregulated in tissue samples from HBV‐positve HCC patients,^26^ consistent with our clinical data. HCC is an inflammation‐related tumour due to HBV/HCB infection, alcohol uptake, and metabolic syndrome. Chronic inflammation is known to cause local tissue hypoxia.^39^ Interestingly, CFL1 was identified to be one of the hypoxia‐responsive gene in HCC cells. HIF‐1α, but not HIF‐2α, was a transcription factor regulating CFL1 expression in HCC cells. Mechanistically, HIF‐1α activated CFL1 transcription by binding to the HRE on the promoter. Therefore, this study provided new insight into the regulatory mechanism involved in CFL1 overexpression in HCC.

A previous study has revealed the promoting role of CFL1 in HCC cell survival and migration.^27^ Here, we also disclosed that CFL1 promoted not only migration, proliferation and invasion, but also EMT in HCC cells and its knockdown repressed tumour extensiveness and metastasis in vivo. Moreover, CFL1 knockdown reversed hypoxia‐induced malignant phenotype of HCC cells. The data mentioned above demonstrate that CFL1 plays an essential role in HCC under a hypoxic microenvironment. A previous study verified that CFL1 interacted with radiation‐sensitive 52 (RAD52) interactor and participates in RAD52‐induced HCC cell proliferation and migration.^25^ Besides, treatment with HDACi induces interaction of CFL1 and Bax's and the mitochondrial translocation of CFL1 to promote cell apoptosis via release of cytochrome C in HCC cells.^27^ In the current study, PLD1 was predicted as a CFL1 interactor using publicly available database. CFL1 positively regulated PLD1 protein rather than mRNA in HCC cells. As the first researchers it was disclosed that CFL1 modulated PLD1 expression at the post‐translational level by repressing the ubiquitin‐mediated proteolysis in HCC cells. Reviewing previous studies, it was demonstrated that PLD1 promotes cell proliferation, migration, invasiveness, in HCC cells AKT and mTOR pathways.^40^ Accordingly, it was found that CFL1 enhanced the AKT pathway activation and EMT via PLD1 in HCC cells. Hypoxia‐induced‐activated AKT signalling and EMT was suppressed by either CFL1 or PLD1 knockdown in HCC cells. Altogether, the CFL1/PLD1 axis played an essential role in hypoxia‐induced HCC progression. As upstream regulator of CFL1, it was first indicated the HIF‐1α‐mediated CFL1 transcription which promoted the CFL1/PLD1/AKT axis in HCC. Direct cytoskeletal targets, such as supervillin,^41^ VASP^10^ and CSRP2,^42^ are known to mediate cancer progression under hypoxic condition. No studies have reported on degradation of PLD1 or activity of E3 ubiquitin ligases,^43^ but we would concentrate on identifying mechanism of regulation of PLD1 ubiquitination in follow‐up studies.

**FIGURE 8:**
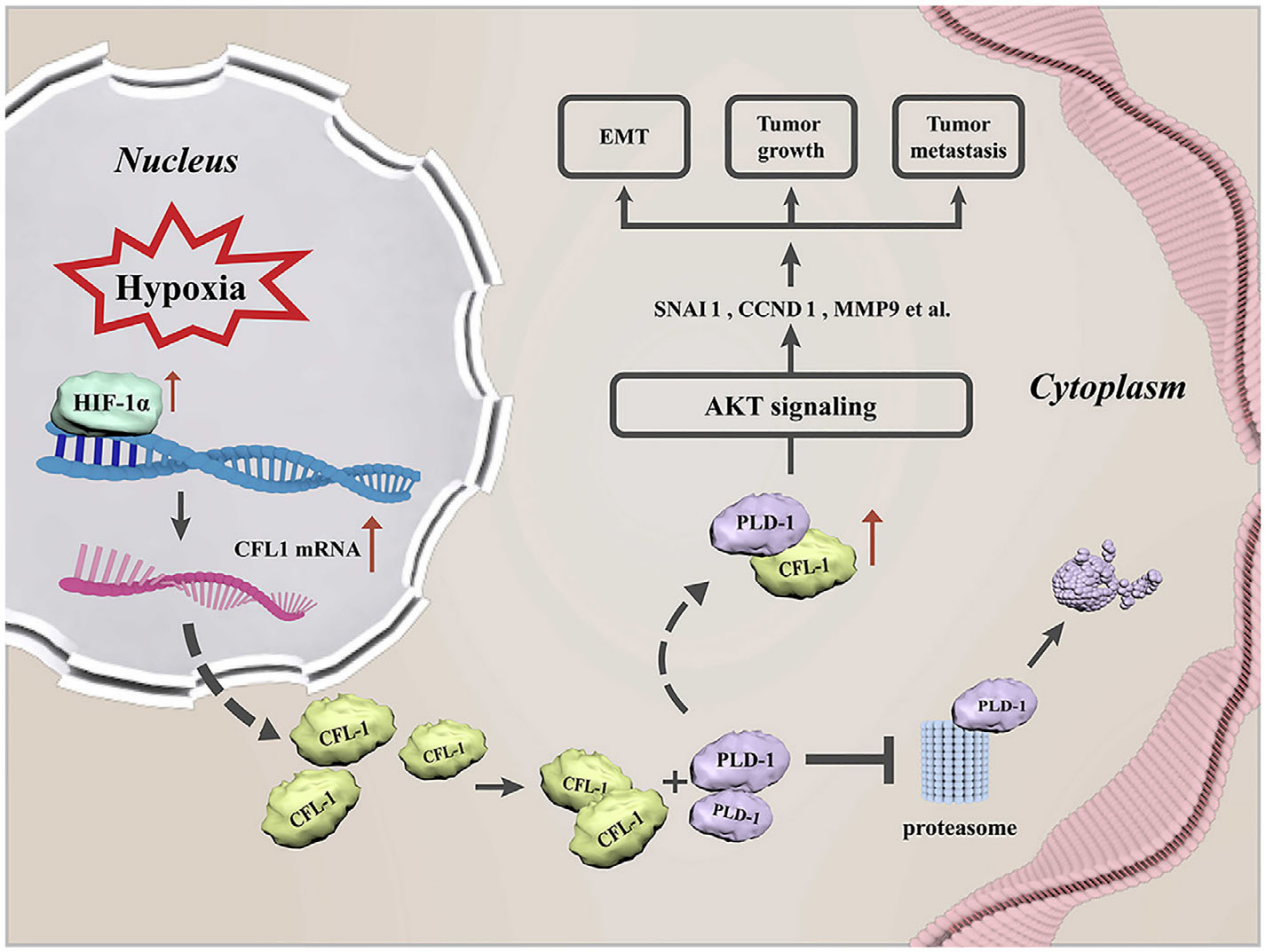
Schematic of the findings of the present study

In conclusion, the analysis has illustrated that over‐expressed CFL1 in HCC was positively associated with poor clinicopathological parameters. In vitro and in vivo, experiments that were performed recognized CFL1 as a driver of tumour growth and metastasis in HCC. The PDL1/AKT pathway mediated the oncogenic functions of CFL1. Furthermore, CFL1 was a hypoxia‐responsive gene and participated in hypoxia‐induced HCC progression by activating PLD1/AKT signalling (Figure 8). Taken together, the study suggests CFL1 as a novel linker between hypoxic microenvironment and HCC progression.

## 5 CONCLUSIONS

In conclusion, the results have revealed the upregulated expression of CFL1 in HCC and its association with poor prognostic and reduced survival in patients. CFL1 enhanced cell growth and metastasis in HCC cells via activation of PDL‐1/AKT signalling. Moreover, it was found that HIF‐1α directly bound to the CFL1 promoter to activate its transcription, and that CFL1 regulated PLD1 expression by repressing its ubiquitin‐mediated proteolysis in HCC cells. To sum up, the study has confirmed that CFL1 plays the role in HCC progression and could be a potential therapeutic target and prognostic predictor in HCC.

## CONFLICT OF INTEREST

The authors declare that they have no competing interests.

## AUTHOR CONTRIBUTIONS

Qingguang Liu and Kangsheng Tu conceived and designed the experiments; Bowen Yao, Yazhao Li, Tiangxiang Chen, Yongshen Niu, Yuanyuan Yang and Xinyu Wei performed the experiments; Bowen Yao and Yazhao Li analysed the data; Yufeng Wang contributed reagents/materials/analysis tools; Bowen Yao, Qingguang Liu and KangshengTu wrote the paper. All authors read and approved the final manuscript.

## Supporting information


**Supplementary Figure 1 The expression of CFL1 in HCC**. (A) TCGA data from the starBase website (http://starbase.sysu.edu.cn/) indicated the upregulated expression of CFL1 mRNA in HCC. (B) GEO dataset (GSE45436) from R2: Genomics Analysis and Visualization Platform (http://r2.amc.nl) confirmed the elevated expression of CFL1 mRNA in HCC. (C) HCC patients with a high CFL1 level had a significantly lower overall survival compared to cases with low CFL1 level. (D) TCGA data analysis using GEPIA webtool revealed that high CFL1 expression predicted reduced overall survival of HCC patients. *p < 0.05.

**Supplementary Figure 2 CFL1 knockdown represses HCC growth and lung metastasis in mice**. (A) MHCC97H cells that were transfected with nontargeting (NT) shRNA or CFL1 shRNAs (shRNA2 and shRNA3) were subcutaneously injected into nude mice. The average tumour volume in the CFL1 knockdown group was prominently smaller than the control group. (B) The tumour weights were compared between the CFL1 knockdown group and the control group. (C) HCCLM3 cells with or without CFL1 knockdown were injected into nude mice via the tail vein. *p < 0.05.

**Supplementary Figure 3** (A) The HRE sequences were found in the promoter of CFL1 based on (Genomatix and JASPAR). (B) Modulating the CFL1 level did not impact PLD1 mRNA expression in HCC cells.

**Supplementary Figure 4 CFL1 knockdown reverses hypoxia‐induced Hep3B cell proliferation and invasion**. (A) Hep3B cells that were transfected with nontargeting (NT) shRNA or CFL1 shRNA were cultured in hypoxic conditions. Western blotting analysis was performed to detect HIF‐1α, CFL1, E‐cadherin, N‐cadherin, and Vimentin levels. (B) CFL1 knockdown repressed the viability of Hep3B under hypoxic conditions. (C) CFL1 silencing abolished hypoxia‐induced Hep3B cell proliferation. (D) The promoting effects of hypoxia on cell migration and invasion were reversed by CFL1 knockdown in Hep3B cells. *p < 0.05.

Supplementary Figure 5 IHC staining of CFL1 and PLD1 was performed in subcutaneous tumour tissues from nude mice.

figurelegends

Supporting Information

Table S1

Table S2
