# Corrigendum to Hypoxia‐induced cofilin 1 promotes hepatocellular carcinoma progression by regulating the PLD1/AKT pathway

**DOI:** 10.1002/ctm2.1110

**Published:** 2022-11-29

Yao, B., Li, Y., Chen, T., et al. Hypoxia‐induced cofilin 1 promotes hepatocellular carcinoma progression by regulating the PLD1/AKT pathway. *Clin Transl Med*. 2021;11:e366. https://doi.org/10.1002/ctm2.366

In the originally published article by Bowen Yao et al. (2021), the authors noted some minor errors in Figure 3D. In Figure 3D, the image of N‐cadherin with shRNA3‐infected treatment was misused from tumor in shRNA2 group. The authors apologize for any inconvenience caused by this error. The corrected figures are given below. The article has been corrected online.

**FIGURE 3:**
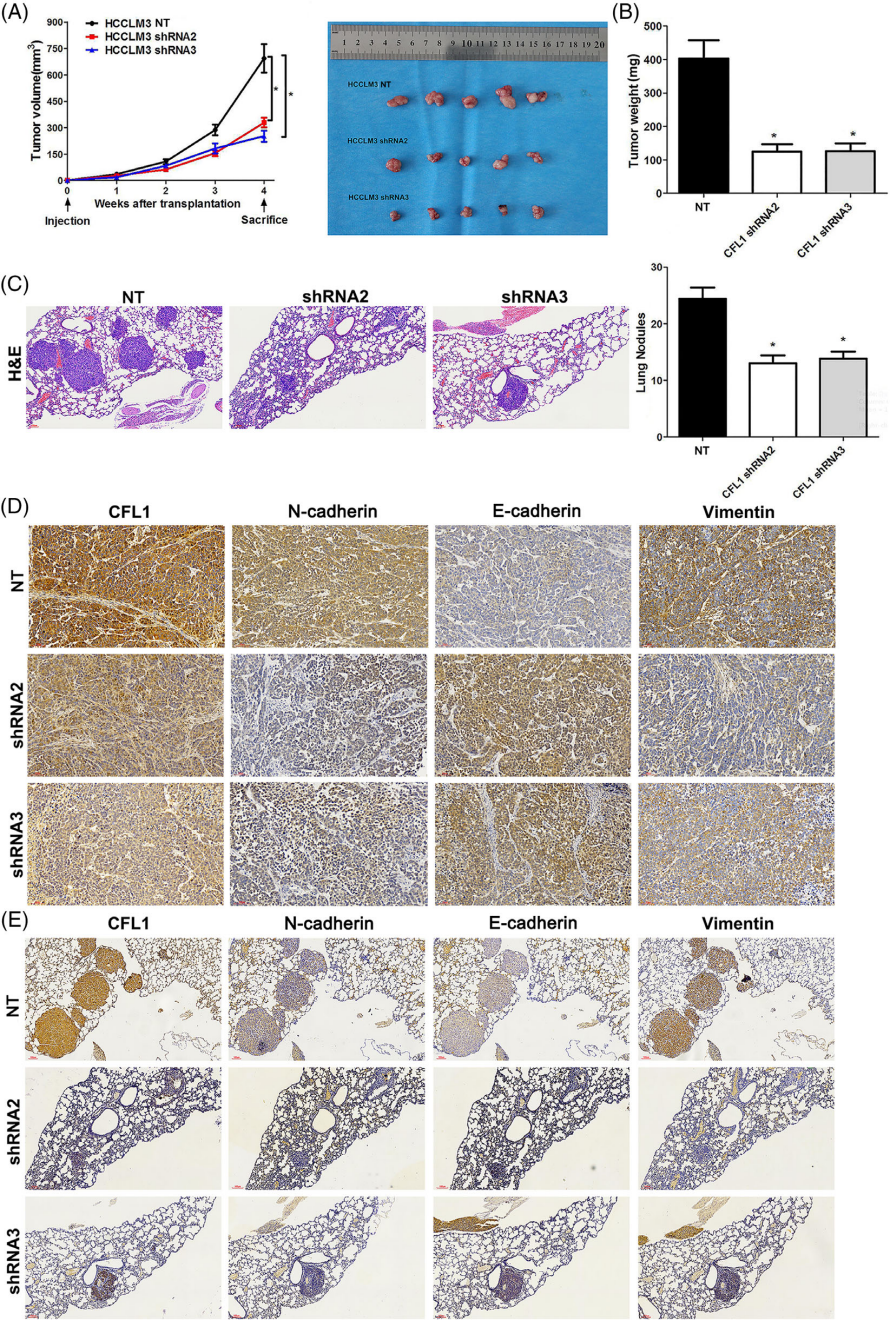
CFL1 knockdown represses HCC growth and lung metastasis in mice. (A) HCCLM3 cells that were transfected with nontargeting (NT) shRNA or CFL1 shRNAs (shRNA2 and shRNA3) were subcutaneously injected into nude mice. The average tumour volume in the CFL1 knockdown group was prominently smaller than the control group. (B) The tumour weights were compared between the CFL1 knockdown group and the control group. (C) HCCLM3 cells with or without CFL1 knockdown were injected into nude mice via the tail vein. H&E staining of lung tissues indicated that CFL1 knockdown reduced lung metastasis of HCC cells. (D) Subcutaneous tumour tissues were subjected to IHC staining for CFL1, E‐cadherin, N‐cadherin, and Vimentin expression. (E) Lung metastases were subjected to IHC staining for CFL1, E‐cadherin, N‐cadherin, and Vimentin expression. *p < 0.05

The correction have no impact on the experimental outcome or conclusions.

