# Supplementary material for: Hypoxia‐induced cofilin 1 promotes hepatocellular carcinoma progression by regulating the PLD1/AKT pathway
Source: Clin Transl Med. 2021 Mar 21;11(3):e366. doi: 10.1002/ctm2.366 (PMC7982636; doi:10.1002/ctm2.366)
Supplement: Supplementary file 6 — figurelegends [file CTM2-11-e366-s004.doc]

Supplementary Figure Legends

**Supplementary Figure 1**

**The expression of CFL1 in HCC.** (A) TCGA data from the starBase website (http://starbase.sysu.edu.cn/) indicated the upregulated expression of CFL1 mRNA in HCC. (B) GEO dataset (GSE45436) from R2: Genomics Analysis and Visualization Platform (http://r2.amc.nl) confirmed the elevated expression of CFL1 mRNA in HCC. (C) HCC patients with a high CFL1 level had a significantly lower overall survival compared to cases with low CFL1 level. (D) TCGA data analysis using GEPIA webtool revealed that high CFL1 expression predicted reduced overall survival of HCC patients. *P<0.05.

**Supplementary Figure 2**

**CFL1 knockdown represses HCC growth and lung metastasis in mice.** (A) MHCC97H cells that were transfected with nontargeting (NT) shRNA or CFL1 shRNAs (shRNA2 and shRNA3) were subcutaneously injected into nude mice. The average tumour volume in the CFL1 knockdown group was prominently smaller than the control group. (B) The tumour weights were compared between the CFL1 knockdown group and the control group. (C) HCCLM3 cells with or without CFL1 knockdown were injected into nude mice via the tail vein. *P<0.05.

**Supplementary Figure 3**

(A) The HRE sequences were found in the promoter of CFL1 based on (Genomatix and JASPAR). (B) Modulating the CFL1 level did not impact PLD1 mRNA expression in HCC cells.

**Supplementary Figure 4**

**CFL1 knockdown reverses hypoxia-induced Hep3B cell proliferation and invasion.** (A) Hep3B cells that were transfected with nontargeting (NT) shRNA or CFL1 shRNA were cultured in hypoxic conditions. Western blotting analysis was performed to detect HIF-1α, CFL1, E-cadherin, N-cadherin, and Vimentin levels. (B) CFL1 knockdown repressed the viability of Hep3B under hypoxic conditions. (C) CFL1 silencing abolished hypoxia-induced Hep3B cell proliferation. (D) The promoting effects of hypoxia on cell migration and invasion were reversed by CFL1 knockdown in Hep3B cells. *P<0.05.

**Supplementary Figure 5**

**IHC staining of CFL1 and PLD1 was performed in subcutaneous tumour tissues from nude mice.**
