# Supplementary material for: Hypoxia‐induced cofilin 1 promotes hepatocellular carcinoma progression by regulating the PLD1/AKT pathway
Source: Clin Transl Med. 2021 Mar 21;11(3):e366. doi: 10.1002/ctm2.366 (PMC7982636; doi:10.1002/ctm2.366)
Supplement: Supplementary file 7 — Supporting Information [file CTM2-11-e366-s006.doc]

**Supplementary Methods**

1. **Real-time quantitative PCR (RT-qPCR)**

TRIzol reagent (Thermo Fisher Scientific) was used to extract total RNA from cells and tissues. Then 1μg total RNA was reverse transcribed to cDNA by RevertAid First Strand cDNA Synthesis Kit (Thermo Fisher Scientific). CFL1, PLD1, and β-actin mRNA levels were detected by BIO-RAD CFX96 (Bio-Rad Laboratories, Hercules, CA, USA) with the SYBR Premix Ex Taq™ II Kit (Takara, Dalian, China). The primers were described in Supplementary Table 1.

1. **Cell proliferation analysis**

2×10^3^ HCC cells in 200μl culture medium were seeded in 96-well plates. At each time point, 20μl of the CCK-8 solution was added into each well, and the plate was incubated for 2h at 37°C. Then the absorbance was measured at 450nm using a Multiskan FC microplate reader (Thermo Fisher Scientific). For EDU staining, EDU Labeling/Detection Kit (Ribobio, Guangzhou, China) was used according to manufacturer’s protocols. Nucleus staining was performed using DAPI, and the proportion of EDU positive cells was calculated after fluorescence microscopy analysis.

1. **Transwell assays**

The Transwell chamber was coated with Matrigel (BD Biosciences, Bedford, MA, USA) for cell invasion assay. HCC cells were suspended in 500μL serum-free medium and added into the inside of the chamber. 800μL of complete DMEM medium was added to the outside of the chamber as a chemoattractant. The Transwell chambers were cultured in an incubator for 24h. The invaded cells were fixed with 10% formaldehyde for 15 min, stained with 0.1% crystal violet for 10 min, and finally photographed under an inverted light microscope with a 40X objective. A Transwell chamber without Matrigel (BD Biosciences) was used for cell migration assay.

1. **Western Blotting**

RIPA lysis buffer (Beyotime) was used for total protein extraction from cells and tissues. The concentration of the protein sample was determined by the Bradford protein assay (Bio-Rad, Hercules, CA, USA). Then, 20μg proteins were separated by 12% SDS-PAGE and then transferred to a 0.22μm PVDF membrane (Millipore, Bedford, MA, USA). The PVDF membranes were blocked with 5% skimmed milk at room temperature for 2 hours and incubated with primary antibodies overnight at 4°C.Primary antibodies: CFL1(sc-53934, Santa Cruz Biotechnology, Dallas, TX, USA), HIF-1α(ab243860, Abcam, Cambridge, MA, USA), AKT antibody(#9272, Cell Signaling Technology, Danvers, MA), phospho-AKT (Ser473) (#4060, Cell Signaling Technology), N-cadherin (#14215, Cell Signaling Technology), E-cadherin (#3195, Cell Signaling Technology), Vimentin (#5741, Cell Signaling Technology), PLD1 (ab68150, Abcam), ubiquitin (ab140601, Abcam), and β-actin (#4970, Cell Signaling Technology). The next day, the PVDF membranes were incubated by HRP-conjugated Affinipure Goat Anti-Mouse IgG (H+L) or Anti-Rabbit IgG (H+L) (BOSTER, Wuhan, China) for 2h at room temperature. The reactive blottings were imaged by GE Amersham Imager 680 (GE Healthcare Life Sciences, Pittsburgh, PA, USA) and quantified by Image J software (National Institutes of Health, Bethesda, MD, USA).

1. **IHC staining**

Primary antibodies including CFL1 (sc-53934, Santa Cruz Biotechnology), PLD1 (ab68150, Abcam), N-cadherin (#14215, Cell Signaling Technology), E-cadherin (#3195, Cell Signaling Technology), and Vimentin (#5741, Cell Signaling Technology) were incubated with sections at 4℃ overnight, following by the second antibody at room temperature for an hour. Finally, tissues were stained by DAB and hematoxylin and then were scanned using the Motic Virtual Microscope System.

1. **Co-immunoprecipitation (co-IP)**

500μg of total protein was extracted from HCC cells for co-IP assay. We used 1μg CFL1 antibody (sc-53934, Santa Cruz Biotechnology) or normal mouse IgG (sc-2025, Santa Cruz Biotechnology) coated the magnetic beads. The detailed procedures of co-IP were performed according to the protocol of Pierce™ Classic Magnetic IP/Co-IP Kit (Thermo Fisher Scientific).

1. **Chromatin immunoprecipitation (ChIP)**

ChIP was performed according to the instruction manual of the EZ-CHIP^TM^ Chromatin Immunoprecipitation Kit (Millipore). Hep3B cells were cultured in normoxic (20% O_2_) or hypoxic conditions (1% O_2_) for 24h. Subsequently, the cells were crosslinked with formaldehyde and sonicated to an average size of 300-500bp. Lysates were immunoprecipitated with HIF-1α (ab243860, Abcam), HIF-1β (Novus Biologicals, Littleton, CO, USA), or normal rabbit IgG (Millipore). Finally, the protein or DNA complexes were reversely crosslinked to free DNA and then later further purified using DNA Spin Columns. The eluted DNA was detected by real-time qPCR using primers listed in Supplementary Table 1.

1. **Luciferase reporter assay**

HEK 293T cells were transfected with pcDNA3.1-HIF1α and pGL3-6xHRE-promoter-luciferase or pGL3-CFL1-promoter-luciferase (Luc, a luciferase reporter construct containing predicted sites) together with pSV40-renilla. Cells were incubated in 1% oxygen condition. Luciferase reporter assay was conducted according to the manufacture’s protocol (Promega). The relative Luc activity was normalized by renilla activity.

1. **Statistical Analysis**

The software of SPSS 21.0 (SPSS, Chicago, USA) and GraphPad Prism 8.0 (GraphPad Software, San Diego, USA) was used for statistical analysis. The data were presented as the mean ± SD of three independent experiments. Moreover, student’s t-test and ANOVA were used to compare different groups (such as data in western blotting, EDU positive rites, tumor size/weight and OD450 in groups). The correlations between CFL1 and clinicopathologic features were compared by the Person chi-square test. The Kaplan-Meier survival analysis and log-rank test were used for determining overall survival. Univariable and multivariable Cox proportional hazards regression were used to analyze the prognostic factors. P <0.05 was considered statistically significant.
