# Supplementary material for: Hypoxia‐induced cofilin 1 promotes hepatocellular carcinoma progression by regulating the PLD1/AKT pathway
Source: Clin Transl Med. 2021 Mar 21;11(3):e366. doi: 10.1002/ctm2.366 (PMC7982636; doi:10.1002/ctm2.366)
Supplement: Supplementary file 8 — Table S1 [file CTM2-11-e366-s001.doc]

**The primer sequences of genes mentioned in our study**

| CFL1 | Forward:5’-GGTGCCCTCTCCTTTTCGTT-3’ |
| --- | --- |
|  | Reverse: 5’-GGCATAGCGGCAGTCCTTAT-3’ |
| PLD1 | Forward: 5’-AAAAGGAGCCACGGGTAAA-3’ |
|  | Reverse: 5’-ATTGGACGGCCGGAGAGA-3’ |
| β-actin | Forward: 5’-CTCGCCTTTGCCGATCC-3’ |
|  | Reverse: 5′-TCTCCATGTCGTCCCAGTTG-3’ |
| HRE | Forward: 5’-TGGAGGAAATGGTGATCCTC-3’ |
|  | Reverse: 5’-TGCCCAAGCACCTCTTACTT-3’ |
| SNAI1 | Forward: 5’-CCTCCCTGTCAGATGAGGAC-3’ |
|  | Reverse: 5’-CCAGGCTGAGGTATTCCTTG-3’ |
| CCND1 | Forward: 5’-GAACCTGGCCGCAATGAC-3’ |
|  | Reverse: 5’-CGCCTCTGGCATTTTGGA-3’ |
| MMP9 | Forward: 5’-ACGCAGACATCGTCATCCAGT-3’ |
|  | Reverse: 5’-GGACCACAACTCGTCATCGTC-3’ |

**The sequences of shRNAs and siRNAs**

| NT shRNA | ACAGAAGCGATTGTTGATC |
| --- | --- |
| CFL1-shRNA1 | GCTGGCCTCTCAAACTTAATG |
| CFL1-shRNA2 | GCTCTACCACTGTTACTTACT |
| CFL1-shRNA3 | GCGTCAGCTCTCTGGAAATGT |
| HIF1ɑ-siRNA | CCAGAUCUCGGCGAAGUAATT |
| NT siRNA | UUCUCCGAACGUGUCACGUTT |
| PLD1-siRNA | UGUAACAGCUACAGACACGTT |
